# Case Report: Frontline CD19 CAR-T-cell therapy combined with targeted regimens induces durable complete remission in newly diagnosed secondary CNS lymphoma: a report of two cases

**DOI:** 10.3389/fonc.2026.1942281

**Published:** 2026-09-16

**Authors:** Qianying Pan, Ran Zhao, Jingyi Yang, Hao Guo, Fenghua Gao, Xingchen Liu, Qing Zhang, Xueli Jiao, Keshu Zhou

**Affiliations:** Department of Hematology, The Affiliated Cancer Hospital of Zhengzhou University & Henan Cancer Hospital, Zhengzhou, Henan, China

**Keywords:** CD19 CAR-T, cell therapy, central nervous system, lymphoma, secondary

## Abstract

Secondary central nervous system lymphoma (SCNSL) at initial diagnosis is an aggressive malignancy associated with a dismal prognosis. Standard-of-care treatments based on high-dose methotrexate often yield suboptimal outcomes. Although CD19-directed chimeric antigen receptor T-cell (CD19 CAR-T-cell) therapy is established in relapsed/refractory settings, its efficacy as a frontline intervention for SCNSL remains poorly defined. We report two cases of treatment-naive SCNSL that achieved sustained complete remission (CR) following a novel sequential therapeutic approach. Case 1 (65-year-old male) received Pola-ZR induction therapy (polatuzumab vedotin, zanubrutinib, and rituximab), while Case 2 (56-year-old female) received ZPR induction therapy (zanubrutinib, pomalidomide, and rituximab) followed by autologous stem cell transplantation. Both patients subsequently received CD19 CAR-T-cell infusions. Rapid CR was achieved and maintained for 24 and 21 months, respectively, with manageable toxicities. Frontline CAR-T-cell therapy combined with targeted agents may constitute a promising therapeutic paradigm for newly diagnosed SCNSL, yet these hypothesis-generating findings require validation in larger patient cohorts.

## Introduction

Secondary involvement of the central nervous system in B-cell lymphoma (SCNSL) at presentation is rare, occurring in <5% of diffuse large B-cell lymphoma (DLBCL) cases (1, 2). Despite intensive chemoimmunotherapy, the 5-year overall survival (OS) remains modest at 20%–56% (3). Current frontline strategies lack consensus and typically rely on high-dose methotrexate (HD-MTX)-based regimens (e.g., MATRix), followed by autologous stem cell transplantation (ASCT) for consolidation (4).

CD19-directed chimeric antigen receptor T-cell (CD19 CAR-T-cell) therapy has revolutionized the treatment of relapsed/refractory (R/R) DLBCL. Recent data from the ZUMA-12 trial highlighted the benefits of frontline CAR-T-cell therapy in high-risk systemic lymphoma; however, patients with CNS involvement were notably excluded (5). Consequently, the feasibility and safety of early CAR-T-cell intervention in SCNSL remain critical clinical questions. Herein, we describe two patients successfully treated with targeted induction followed by frontline CD19 CAR-T-cell therapy.

## Case presentation

Case 1: A 65-year-old male presented with dizziness in April 2024. Neuroimaging showed a cystic and solid space-occupying lesion in the posterior horn of the right lateral ventricle adjacent to the corpus callosum, with obvious enhancement, central liquefaction necrosis, and peritumoral edema (**Figure 1B**). Whole-body PET/CT imaging showed active metabolism in the soft tissue shadow of the cystic and solid space-occupying lesion in the posterior horn of the right lateral ventricle adjacent to the corpus callosum, with a maximum standardized uptake value (SUVmax) of approximately 31.8 and a size of approximately 2.0 × 3.1 cm. Local bone density reduction and active metabolism were observed in the right acetabulum, with a SUVmax of approximately 22.5, indicating infiltration (**Figure 1C**). Due to the specific nature of the lesion in the right acetabulum, it was considered that obtaining pathological samples would be challenging. Subsequently, an intracranial space-occupying lesion biopsy was performed, and postoperative pathology revealed DLBCL, non-GCB subtype. The Eastern Cooperative Oncology Group (ECOG) Performance Status (PS) score was 1, whereas the Karnofsky Functional Status (KFS) score was 80.

**Figure 1:**
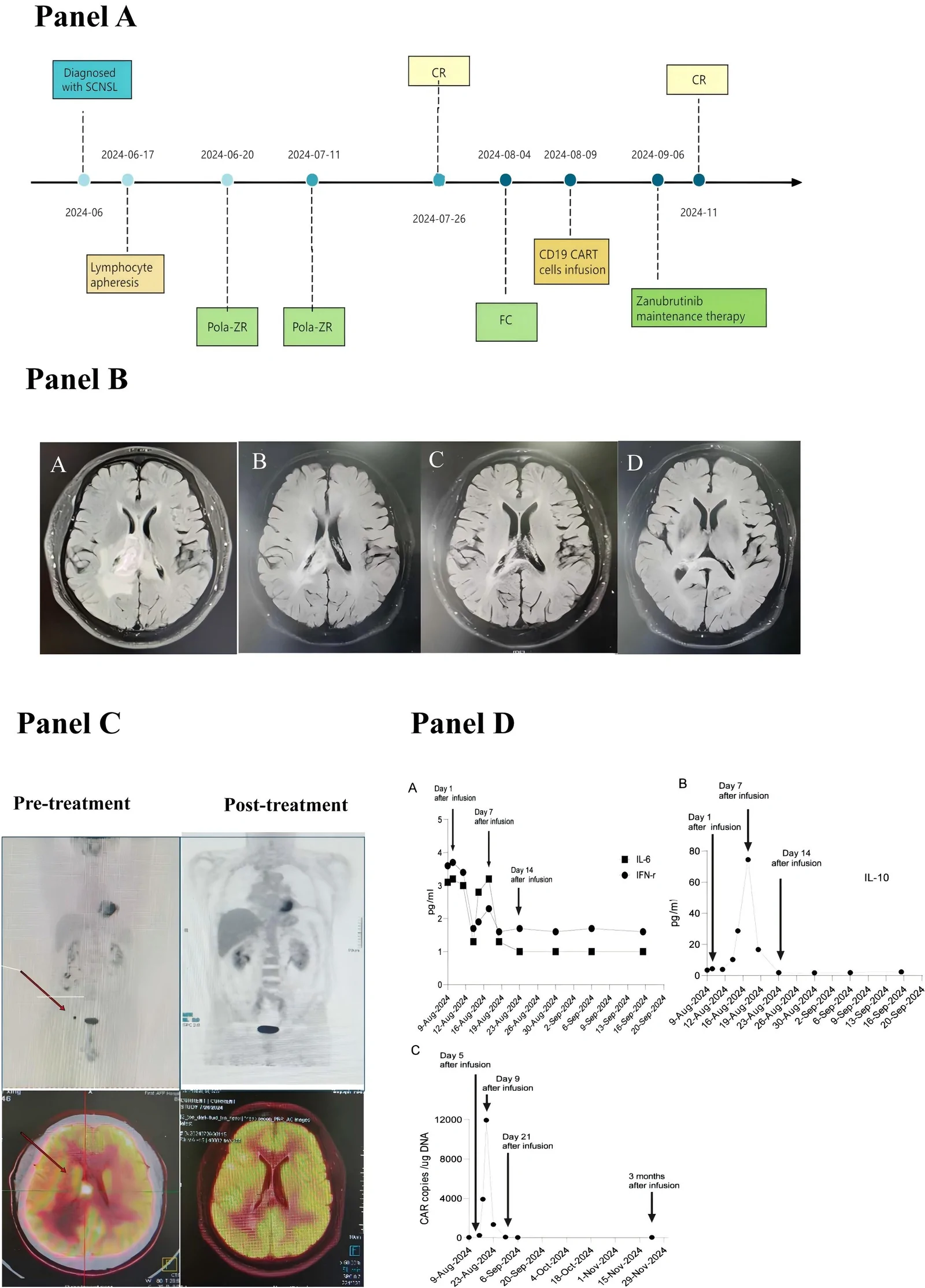
Clinical and radiologic characteristics of Case 1 patient. Panel **A** shows the timeline of treatment process in Case 1 **B** shows the brain MRI images of the lesion site at different stages in Case 1 **(A)** Pretreatment image **(B)** Image after two courses of induction therapy **(C)** Image after 3 months of CD19 CAR-T-cell therapy **(D)** Image after 14 months of CD19 CAR-T-cell therapy **C** shows whole-body PET/CT images before and after CD19 CAR-T cell therapy in Case 1 **D** shows changes in cytokine levels and CAR-T-cell copy number in peripheral blood after CD19 CAR-T-cell infusion in Case 1 **(A)** Trend chart of IL-6 and IFN-r levels after infusion **(B)** Trend chart of IL-10 levels after infusion **(C)** Changes in CAR-T-cell copy number after infusion .

Immunohistochemistry showed AE1/AE3 (−), LCA/CD45 (+++), CD3 (−), CD20 (+++), CD19 (++), GFAP (−), CD30 (−), CD56 (−), CD10 (−), Bcl-6 (+), MUM1 (+), Bcl-2 (60%+), C-Myc (20%+), Ki67 (80%), and EBER (−). FISH showed no BCL-2 or C-MYC gene breakage. Pathological diagnosis was DLBCL, non-GCB subtype. Tumor tissue gene subtype was detected as MCD subtype through second-generation sequencing. MYD88 (p.L265P) was found to have predictive value for drug efficacy. Laboratory tests showed normal serum LDH levels, negative bone marrow evaluation, and elevated cerebrospinal fluid (CSF) protein without malignant cells.

The final diagnosis was DLBCL (stage IV, non-GCB subtype, with CNS and bone involvement; IPI score: 3). Given the high-risk features with initial CNS involvement, CD19 CAR-T-cell therapy was selected after shared decision-making. Lymphocyte apheresis was performed on June 17, 2024. Starting on June 20, 2024, the patient received the Pola-ZR regimen consisting of rituximab (375 mg/m^2^ on day 1), polatuzumab vedotin (1.8 mg/kg on day 2), and zanubrutinib (160 mg twice daily). Each 21-day period constituted one treatment course. During induction chemotherapy, intrathecal prophylaxis was administered for CNS involvement. Supportive care was provided for mild upper respiratory infection and liver dysfunction during treatment.

After two courses, whole-body PET/MR performed on July 26, 2024, revealed resolution of metabolic activity and reduction in lesion size in the brain and right acetabulum. The treatment response was evaluated as complete remission (CR).

On August 4, 2024, the patient received lymphodepletion pretreatment with the FC regimen, consisting of fludarabine (30 mg/m^2^ on days 1–3) and cyclophosphamide (500 mg/m^2^ on days 1 and 2). The procedure was completed smoothly, and on August 9, 2024, he received CD19 CAR-T-cell infusion. On day +6 after CAR-T-cell infusion, he developed fever, which was considered to be associated with COVID-19 infection. His temperature was controlled with active anti-infective treatment. During this period, mild cytokine reaction and good CAR-T-cell expansion were observed (**Figure 1D**). On day +28 after CAR-T-cell infusion, disease assessment showed stable disease, and zanubrutinib (160 mg twice daily) was added as maintenance therapy. Three months after CAR-T-cell infusion, whole-body PET/MR scan was performed, which indicated no significant increase in abnormal signal metabolism in the right posterior horn of the lateral ventricle or the pericallosal region, with a slightly reduced range compared with that observed in previous scans. No abnormal signal metabolism was observed in the right acetabulum, and the treatment response was assessed as CR.

The patient has been followed up to date, and the general condition is good with a continuous CR status. The patient continues to receive zanubrutinib for maintenance therapy. From April 2024 to April 2026, both progression-free survival (PFS) and OS were 24 months (**Figure 1A**).

Case 2: A 56-year-old female presented with dizziness, headache, and nausea in July 2024. Imaging revealed a blood-rich space-occupying lesion in the right temporo-occipital lobe, pituitary stalk, and left suboccipital gyrus (**Figure 2B**). Whole-body PET/CT showed slightly high-density nodules with active metabolism in the right parieto-occipital lobe, right occipital lobe, and pituitary stalk, with SUVmax of 52.8 and a lesion size of approximately 2.3 × 2.7 cm. Soft tissue nodules with active metabolism were also observed in the right adrenal gland, with a SUVmax of 41.4 and a lesion size of approximately 1.2 × 1.5 cm (**Figure 2C**). PET/CT findings revealed that the nature of the intracranial and adrenal space-occupying lesions was uncertain; therefore, an adrenal biopsy was planned. However, owing to the atypical imaging appearance of the adrenal lesions and the difficulty in obtaining a biopsy sample, intracranial biopsy was recommended. On August 8, 2024, a transoccipital craniotomy was performed. Postoperative pathology revealed invasive B-cell lymphoma. The ECOG PS score was 1, and the KFS score was 70.

**Figure 2:**
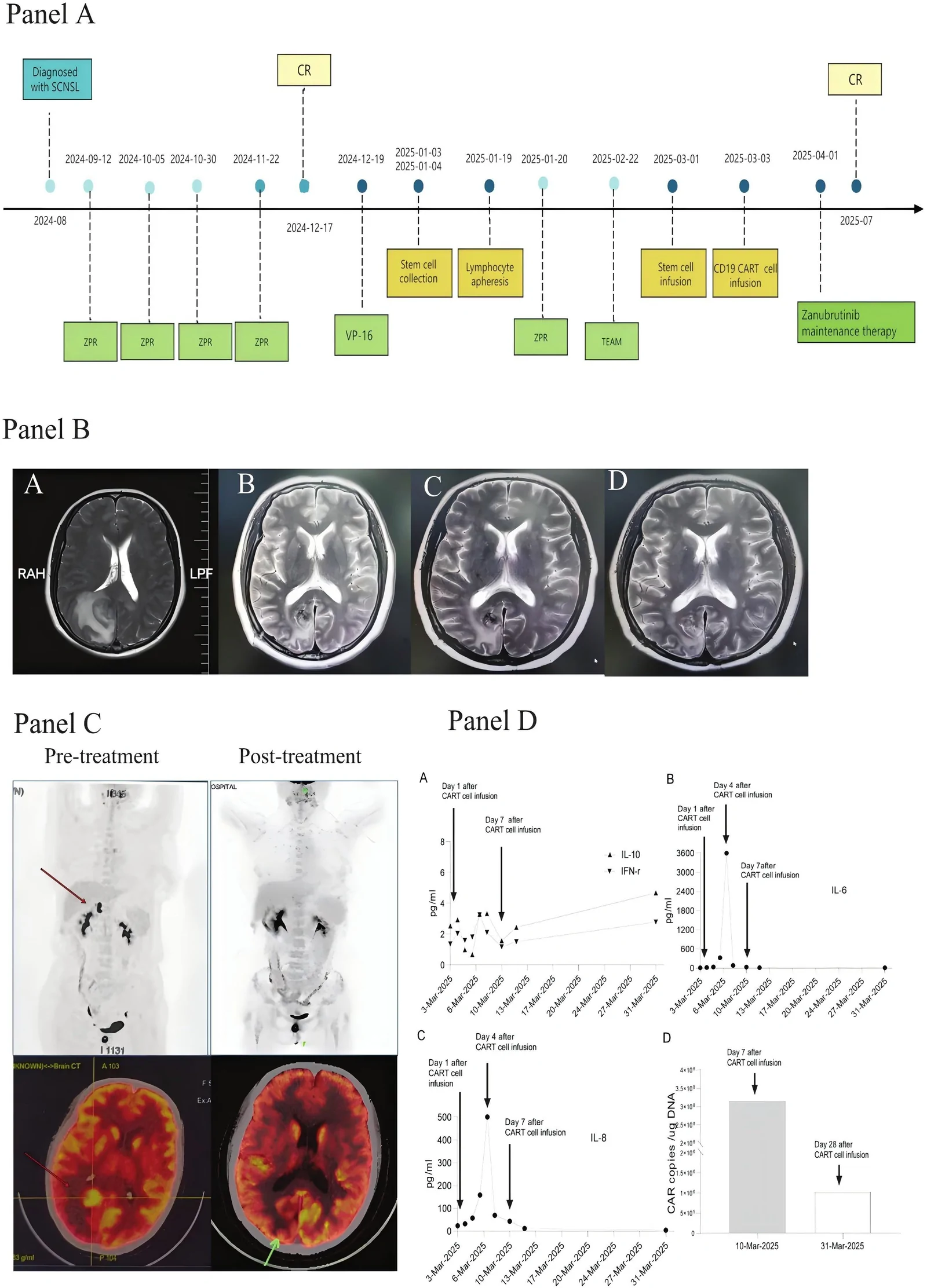
Clinical and radiologic characteristics of Case 2 patient. **A** shows the timeline of treatment process in Case 2 **B** shows the brain MRI images of the lesion site at different stages in Case 2 **(A)** Pretreatment image **(B)** Image after two courses of induction therapy **(C)** Image after 3 months of CD19 CAR-T-cell therapy combined with ASCT **(D)** Image after 9 months of CD19 CAR-T-cell therapy combined with ASCT **C** shows whole-body PET/CT images before and after CD19 CAR-T cell therapy in Case 2 **D** shows changes in cytokine levels and CAR-T-cell copy number in peripheral blood after CD19 CAR-T-cell transfusion in Case 2 **(A)** Trend chart of IL-10 and IFN-r levels after infusion **(B)** Trend chart of IL-6 levels after infusion **(C)** Trend chart of IL-8 levels after infusion **(D)** Changes in CAR-T-cell copy number after infusion.

Immunohistochemical analysis showed AE1/AE3 (−), GFAP (−), CD20 (+), CD79a (+), CD19 (diffusely strong+), CD3 (−), CD43 (−), CD21 (+), CD5 (partially weak+), CyclinD1 (−), CD10 (weak+), Bcl-6 (approximately 30%+), Bcl-2 (approximately 40%+), MUM-1 (+), CD30 (−), Ki-67 (approximately 80%+), P53 (approximately 80% moderately-strong+), c-Myc (approximately 60%+), and EBER (−). FISH demonstrated 11q23.3 amplification and 11q24.3 deletion, while BCL-2, BCL-6, and C-MYC showed no gene breakage. The pathological diagnosis was high-grade B-cell lymphoma (HGBL) with 11q abnormality. Tumor tissue second-generation sequencing identified a MYD88 (p.L265P) gene mutation, corresponding to the molecular MCD subtype. Serum LDH was elevated, bone marrow examination was negative, and CSF findings were normal.

The final diagnosis was HGBL (with 11q abnormality, stage IV, involving the CNS and right adrenal gland; MCD subtype; IPI score: 3). On September 12, 2024, the patient started the ZPR regimen consisting of rituximab (375 mg/m^2^ on day 1), pomalidomide (4 mg once daily), and zanubrutinib (160 mg twice daily), with each 21-day cycle constituting one treatment course. During induction chemotherapy, lumbar puncture and intrathecal therapy were administered for CNS involvement. Upper respiratory tract infection and mild liver dysfunction were managed with supportive care. After four courses, follow-up whole-body PET/CT scan performed on December 17, 2024, showed patchy slightly low-density shadows with reduced radioactive uptake in the surgical area. Local cortical discontinuity with nodular high-density shadows was observed in the right skull, but no abnormal radioactive uptake was detected. Slightly increased metabolism in the right adrenal gland was considered a post-treatment change. The treatment response was assessed as CR.

Given the CNS involvement at onset and high-risk disease features, ASCT combined with CD19 CAR-T-cell therapy was planned. Mobilization chemotherapy with VP-16 (etoposide 300 mg/m^2^ on days 1–3) was initiated on December 19, 2024, and the process went smoothly. G-CSF was administered starting on December 25, 2024, for stem cell to mobilization. Autologous hematopoietic stem cell collection was performed on January 3 and 4, 2025, with a nucleated cell count of 5.92 × 10^8^/L and a CD34+ cell count of 8.11 × 10^6^/L. On January 19, 2025, peripheral blood lymphocytes were collected for CAR-T-cell preparation, and an adequate cell count was obtained. On January 20, 2025, the patient received bridging therapy with the ZPR regimen at the same dosage as before, and the process went smoothly.

On February 22, 2025, the TEAM regimen (thiotepa 8 mg/kg qd1, cytarabine 400 mg/m^2^ qd2–5, etoposide 200 mg/m^2^ qd2–5, and melphalan 140 mg/m^2^ qd6) was initiated as pretreatment. On March 1, 2025, autologous hematopoietic stem cell infusion was performed. On March 3, 2025, CD19 CAR-T cells were infused. On day +4 after stem cell infusion and day +2 after CAR-T-cell infusion, the patient developed fever, which was considered to be owing to cytokine release syndrome (CRS). Low-dose steroids were administered, and anti-infection treatment was actively pursued. On day +5 after stem cell infusion and day +3 after CAR-T-cell infusion, the patient experienced severe abdominal pain. Emergency abdominal CT scan revealed no significant abnormalities, and a high possibility of intestinal infection was considered. Treatment involving fasting, somatostatin administration, and mucosal repair promotion improved the symptoms. On day +11 after stem cell infusion, granulocyte engraftment occurred. During this period, mild elevation of cytokines was monitored, and CD19 CAR-T-cell expansion was good (**Figure 2D**).

After the infusion of CAR-T cells, the disease efficacy was assessed as stable at +28 days, and the patient was maintained on treatment with additional zanubrutinib (160 mg twice daily). Three months after CD19 CAR-T-cell infusion, follow-up whole-body PET/CT scan revealed multiple low-density lesions in the right occipital lobe with enlargement of the adjacent right posterior lateral ventricle but with low metabolism and no activity in the local lesions. The right adrenal gland was normal. The treatment response was assessed as CR.

The patient has been followed up to date, and the general condition is good with a continuous CR status. The patient continues to receive zanubrutinib for maintenance therapy. From July 2024 to April 2026, both PFS and OS were 21 months (**Figure 2A**).

## Discussion

SCNSL is defined as involvement of the CNS occurring either at the initial diagnosis or at recurrence of systemic lymphoma. It may present as isolated CNS disease or simultaneously with systemic involvement. Newly diagnosed SCNSL accounts for approximately 40% of all SCNSL cases (6). The management of newly diagnosed SCNSL is historically challenging because of poor drug penetration across the blood–brain barrier (BBB) and high relapse rates. Currently, there is no unified international recommendation for the treatment of SCNSL, and most reported regimens are based on HD-MTX. The MARIETTA single-arm phase II clinical trial initiated in Europe is the largest prospective study of SCNSL to date. It included 32 patients with newly diagnosed SCNS DLBCL, 28 patients with recurrent SCNS DLBCL accompanied by systemic involvement, and 15 patients with recurrent SCNS DLBCL without systemic involvement. Patients received three courses of MARTix and three courses of RICE, followed by ASCT consolidation therapy with carmustine combined with thiotepa-based conditioning. The optimal ORR for initial treatment of SCNS DLBCL was 75%, CR rate was 55%, and 1-year PFS was 75% (4). A clinical study from the UK included 10 patients with newly diagnosed SCNS DLBCL who received an alternating regimen of R-CODX-M and R-IVAC, resulting in a 2-year PFS of 70% (7). Similarly, the R-MTX-CHOP regimen showed comparable efficacy, with a 3-year PFS of 56% (2). However, chemotherapy-related toxicity remains substantial. CD19 CAR-T-cell therapy is another potential option for SCNSL; however, to date, it has only been used in R/R cases and has not yet been routinely used as first-line treatment (8).

The use of CAR-T-cell therapy as first-line treatment for high-risk B-cell lymphoma, particularly in patients with CNS involvement, provides a novel treatment approach. The ZUMA-12 study aimed to explore the application of CAR-T-cell therapy as first-line treatment for high-risk DLBCL patients, defined as those with MYC and BCL2 and/or BCL6 rearrangements (double/triple impact lymphoma) or those with an IPI score ≥3 and with mid-term PET positivity after two courses of chemotherapy or immunotherapy. In the ZUMA-12 study, the CR rate of first-line high-risk DLBCL patients receiving CAR-T-cell therapy reached 86%, and the 3-year OS rate was 81%, indicating that earlier administration of CAR-T-cell therapy may provide greater clinical benefit (5). However, the ZUMA-12 study did not yet include patients with CNS involvement. CAR-T-cell therapy has shown encouraging efficacy and acceptable safety in patients with CNS lymphoma (9). A group of eight patients with refractory SCNSL who received CD19 CAR-T-cell therapy showed no significant toxicity, with a CR rate of 50% (10). A recent clinical study involving 100 patients with R/R PCNSL and SCNSL treated with CD19 CAR-T-cell therapy reported 24-month OS and PFS rates of 37% and 28%, respectively. In that study, 83% and 42% of patients developed any grade of CRS and immune effector cell-associated neurotoxicity syndrome (ICANS), respectively. Overall, 11 patients developed grade 3–4 CRS and 17 patients developed grade 3–4 ICANS (11). Both high-risk patients in our report safely and effectively received first-line CD19 CAR-T-cell therapy, avoiding chemotherapy toxicities and improving quality of life. The application prospects of CAR-T-cell therapy in SCNSL are broad, but its neurotoxic effects, particularly CRS and ICANS, limit its research and application in patients with SCNSL (9). Both patients experienced mild CRS reactions throughout the CD19 CAR-T-cell treatment and did not show signs of ICANS upon careful monitoring. These mild adverse reactions in these patients are potentially attributable to their good response to previous treatments and low tumor burden. These two cases demonstrate that in CNS-involved lymphoma, CAR-T-cell therapy can achieve high remission rates with favorable safety when toxicities are well managed.

Polatuzumab vedotin (Pola), a monoclonal antibody targeting CD79b coupled with the microtubule inhibitor MMAE, has demonstrated antitumor activity in B-cell lymphoma (12). The POLARIX trial, a randomized double-blind phase III multicenter clinical trial conducted in Europe, included 879 patients with DLBCL. The results showed that Pola-R-CHP treatment for untreated DLBCL significantly improved prognosis compared with the R-CHOP regimen (2-year PFS: 76.7% vs. 70.2%, *p* = 0.02) (13). Currently, the treatment regimen containing Pola has been recommended by treatment guidelines as first-line therapy for patients with DLBCL. The fully targeted Pola-ZR regimen offers high efficacy, minimal toxicity, and is relatively friendly to elderly patients (14). However, the efficacy of Pola in SCNSL remains unclear, as the POLARIX study excluded patients with CNS involvement. In Case 1, two courses of the Pola-ZR regimen achieved rapid disease control and successfully bridged the patient to CD19 CAR-T-cell therapy, indicating that the Pola-ZR regimen may penetrate the BBB and effectively target CNS lymphoma.

Small-molecule agents, such as BTK inhibitors and immunomodulatory drugs, have also shown activity as monotherapy in R/R PCNSL. Their favorable CNS bioavailability suggests that, when combined with the R-CHOP regimen, they prevent CNS recurrence. BTK inhibitors, such as ibrutinib, have shown encouraging activity in patients with PCNSL and abundant MYD88 and CD79 mutations. In a phase II study of 44 patients with R/R CNS lymphoma, the ORR for patients with SCNSL and PCNSL receiving ibrutinib treatment were 69% and 81%, respectively, with a median PFS of 4 months (15). Ibrutinib has also been used in combination with MTX and rituximab, achieving encouraging results (16). Second-generation BTK inhibitors, such as zanubrutinib, may cross the BBB more efficiently and have demonstrated good efficacy in patients with CNS lymphoma. Immunomodulatory drugs, such as lenalidomide and pomalidomide, have also been used alone or in combination with rituximab for R/R PCNSL, with reaction rates of approximately 50%. A study of lenalidomide and rituximab in 14 patients with R/R CNS lymphoma reported that 3 out of 8 SCNSL patients experienced reactions (17). Pomalidomide, a new-generation immunomodulator, demonstrates better efficacy and has fewer side effects. In Case 2, four courses of the ZPR regimen were used as induction therapy, which effectively controlled the disease and enabled subsequent CD19 CAR-T-cell infusion. This supports that ZPR regimen, containing small-molecule agents, can cross the BBB and effectively treat CNS lymphoma. Both patients received zanubrutinib maintenance therapy, which further consolidated efficacy with favorable safety.

No prospective randomized trials have compared ASCT with other consolidation therapies for SCNSL. However, other studies have reported that ASCT consolidation therapy significantly improved the prognosis of patients with newly diagnosed SCNS DLBCL (3-year OS: 75% in the ASCT group vs. 29% in the non-ASCT group) and reduced the risk of recurrence, rendering ASCT a standard treatment for SCNS DLBCL (18). In PCNSL, prognosis of ASCT was better with the thiotepa-containing pretreatment regimen than with BEAM (19). In Case 2, the patient was treated with the TEAM regimen pretreatment followed by ASCT + CD19 CAR-T-cell therapy. For patients with R/R DLBCL, ASCT + CAR-T-cell therapy has been associated with higher CR rates, longer PFS, and lower recurrence/progression rates (20). In another real-world study involving 171 DLBCL patients, the CR rate in the ASCT + CAR-T-cell group was significantly higher than that in the CAR-T-cell group. Compared with the CAR-T-cell group, the ASCT + CAR-T-cell group also demonstrated better OS, and the PFS benefit persisted over time. ASCT + CAR-T-cell did not increase non-recurrent mortality, and the incidence of grade 3 CRS and ICANS was comparable to that observed with CAR-T-cell therapy alone. Compared with CAR-T-cell therapy alone, ASCT + CAR-T-cell therapy may improve survival and response rates (21). Reports of ASCT + CD19 CAR-T-cell as frontline treatment for high-risk B-cell lymphoma remain limited. Pre-transplant conditioning not only clears the patient’s immune cells and reduces tumor burden, but also mitigates the risk of CRS during subsequent CAR-T treatment. Meanwhile, the infusion of autologous hematopoietic stem cells provides hematologic support, thereby reducing the risk of bone marrow suppression after CAR-T therapy (22). Case 2 in this study received this first-line regimen with mild and manageable toxicity.

In this report, both patients with newly diagnosed SCNSL achieved CR after receiving CD19 CAR-T-cell therapy. Both patients remain in the maintenance treatment phase and have demonstrated sustained CR on multiple follow-up evaluations. The CD19 CAR-T-cell therapy is recommended as a first-line treatment option for the initial treatment of SCNSL. The prognosis of initial treatment of SCNSL patients is significantly better than that of those with R/R SCNSL. Aged over 60 years, ECOG PS >1, simultaneous involvement of brain parenchyma and meninges, and poor response to induction therapy are adverse prognostic factors for SCNSL (23). Both patients with newly diagnosed SCNSL had an ECOG PS score of 1 and only isolated brain parenchymal involvement. After induction therapy, CR was immediately achieved, representing a favorable prognostic factor. The treatment plan adopted by these two patients was safe and effective, with few side effects, long duration of CR, and high quality of life for these patients. Frontline use of CD19 CAR-T-cell therapy for SCNSL provides new hope for patients.

Although our retrospective study demonstrates favorable imaging and survival outcomes following CAR-T-cell therapy for CNS lymphoma, objective endpoints cannot fully capture patients’ real-world experiences. CNS-targeted CAR-T is associated with unique disease- and treatment-related burdens, including transient or persistent neurocognitive dysfunction, memory impairment, severe fatigue, recurrent infections, psychological distress, and long-term fear of recurrence (24). As a non-invasive modality with high sensitivity and specificity, ctDNA-based liquid biopsy enables real-time assessment of tumor burden and treatment responses in CNS lymphoma (25). Unfortunately, plasma and CSF ctDNA monitoring was not available for our two patients. Integration of ctDNA testing may facilitate diagnosis, disease surveillance, and prognostic evaluation in future clinical practice. This study has several limitations. First, as a single-center retrospective case series with a small sample size, potential selection bias exists, and the generalizability of our findings is limited. Second, insufficient long-term follow-up precludes evaluation of the long-term efficacy and safety of CAR-T. Third, standardized questionnaire-based quality-of-life and patient-reported outcome assessments were absent, which precluded quantitative evaluation of neurocognitive function, subjective symptoms, psychological burden, and daily-life status. Larger-scale investigations are warranted to validate our observations.

## Data Availability

The original contributions presented in the study are included in the article/supplementary material. Further inquiries can be directed to the corresponding author.
